# Diagnosis and Treatment in Dental Prosthesis

**Published:** 1914-07-15

**Authors:** Ellison Hillyer


### DIAGNOSIS AND TREATMENT IN DENTAL PROSTHESIS.

BY ELLISON HILLYER, D.D.S. Sc.D.

Extract from a paper read before the Ontario Dental Society and published in the May Dominion Journal.

When a case demanding prosthetic operation presents there are many things which should be carefully noted before the first step in actual procedure is taken. First, those demanding partial prosthesis; the general condition and health of the various parts of the oral cavity and the teeth; the presence of roots to be saved or extracted; the accumulation of calculus to be removed; the excess gum tissue to be removed or displaced; the presence ot hard parts and elevations. The muscular attachments, so prone to cause subsequent trouble, if not properly dealt with at the beginning. Each and every one of these must be considered and procedure carefully planned with them in view if we can reasonably expect to make any definite prognosis.

Many times our initial examination will indicate systemic conditions of which the patient is not even aware. Cooperation at once, in a confidential way, should be established with the physician under whose habitual care the patient has been. The safety of the operator himself greatly depends upon this. Several cases of this character have come under my observation. The saving of roots and isolated teeth should receive the closest attention. The removal of the various deposits on gingival areas of the teeth is so often grossly neglected that the subsequent restorations fail to meet their fullest efficiency. The failure to relieve hard areas—except in the most conventional way has formed a serious drawback to the success of many an operation. The comparative ease with which the excessively low attachment of the various contiguous muscles may be resected, makes it possible to provide wider areas of firm ridge upon which to rest the denture.

In the edentulous case the observance of the hard and soft parts is vitally important. A very interesting and, to me, valuable addition to this line of thought was brought to my attention by Dr. Ruyl, of New York. A few years ago, the doctor had occasion while abroad to visit one of the Krupp foundries, where the care of the health of the employees includes medical supervision. When necessity demands the removal of all the teeth remaining in an arch, the surgical operation is there carried to the extent of the removal of the entire alveolar processes and superfluous gum tissue. The result of this treatment renders it possible to restore the masticatory apparatus as soon as the tissues heal—usually within one month. The object in taking this direct route to the most permanent condition of ridge to be obtained lies in the conservation of the resources of both employer and employee by providing as soon as possible a permanent denture, thus making it possible for the employee to do his best work under improved conditions with the least loss of time and expense. This, of course, may be considered somewhat radical treatment, in our enlightened circles of practice, but it has led Dr. Ruyl, and it will no doubt lead others to treat all cases where excessive tissue is present in a similar manner. All of us have, from time to time, been annoyed by cases where loose flabby gum tissue is present, not only on the ridge areas, but, in some cases, extending in mushroon appearance over the entire vault. A denture for such a condition, made after the most approved methods, will still be unstable even while retaining its adhesion to the soft tissues. By locally anaesthetizing the parts to be removed, all this soft area may be readily and expeditiously removed, and in a few days the portion to be covered by the denture will present a firm surface.

The selection of an impression material is a very much debated point at the present time. Some consider that no impression should be taken in anything other than plaster of Paris. Others take the stand in defense of modelling compound; there is good reason to believe both have their fields of usefulness. The writer has always placed his dependence upon plaster and has found that it is not as stable as he could wish. That the Greene method of taking modelling compound impressions of edentulous cases, using the Kerr impression trays and allowing the tissues of the mouth to assume their natural positions during the operation of the taking of the impression, is a good one, the writer is assured by the comparatively few tests made thus far. Yet, plaster still has a wide field of usefulness, especially in partial cases, where the truest results may be obtained, if care be used.

In such cases, the tray being removed from the plaster—which has thoroughly set in the mouth—the impression should be removed in sections and assembled, either in the tray or independent of it, and the sections united with hard wax.

The plaster cast, we all know by experience, is decidedly unstable. Indeed, it seems to have been proven conclusively that the cast is constantly changing in form. A remedy for this would be found were it possible to make a tin die as soon after taking the impression as possible. Experiments are under way at present whereby a deposit of any desired thickness may be made directly on the surface of the impression by means of a spraying device. If this is as successfully accomplished as it has been in attempts in other fields we may shortly be provided with a means of obtaining a stable model upon which to vulcanize.

Probably there is no greater field for improvement than in the selection and treatment of porcelain teeth. The blame for the present condition has been so generally placed upon the manufacturers that the majority of the profession probably believe that they are at fault. This is decidedly untrue and no thoughtful practitioner would hesitate to acknowledge that the general demand has created a heterogeneous supply from which we are expected to select the nearest approach to the required shape, size and shade. We have of late been assured that we will soon have at our disposal a definite number of moulds of teeth based upon a scientific classification of three distinct types of teeth in varying sizes and shades. The work which Dr. J. Leon Williams has been doing along this line gives promise of a very much more reasonable selection than any now at our disposal. Yet, given the best possible oppurtunities for selection, there will inevitably remain the necessity for treatment of the teeth for each individual case. Edentulous restorations, in ninety per cent. of cases, are for patients of at least fifty years of age. The exceptions may easily be placed in the liberal allowance of ten per cent.

No teeth for patients of advanced years could be expected to present the finished contour and perfect outline forms which are furnished in the porcelain teeth at present supplied or those which are planned. Nor would it be reasonable to expect any stock to contain the unlimited variety of teeth which are necessary to meet the demand of a natural restoration. No better treatment can be given the present porcelain teeth than that furnished by the application of either the mineral stains, or the oil paints used for the decoration of china. It is conceded by all that there is a necessity for the selection of at least three shades of teeth in any given case. Dr. Hunt, of Omaha, has stated that eight shades may be easily found in the various parts of the average arch. If this be so, it behooves us to spend just a little additional time upon these teeth with results which will satisfy not only ourselves but our patients, and, more than that, the patient's family and freinds, all of whom are on the "qui vive" to criticise, favorably or unfavorably, upon appearances.

Anatomical articulation has been taking great strides forward within the last ten years. Basing their deductions upon the work laid down by Bonwill, Walker, Christiansen and others, Drs. Gritman, Snow and Gysi have placed in our hands articulators which, especially in the latter, leave little to be desired. A close attention to details in the taking of wax occlusion, the use of a trial base plate of the Graft type, the proper transfer of the finished wax occlusion from the mouth to the articulator by means of either the Snow Face-bow or the Gysi Condyle Path Register, will furnish a result only appreciated by those who have followed the technic. The setting up of the teeth becomes a much simpler procedure and the result much more certain than by the former method of "hit or miss" articulation.

What sins have been laid at our doors through the years of faulty bridge construction!

How great a lack of proper diagnosis has been apparent in many cases which are constantly presenting.

The patients have been, to a degree, responsible, however, for most of them have not been educated up to the point of appreciating that all removable dentures are not to be rejected. The sentiment against removable pieces has been very general, but fortunately, in the light of present day methods, it is growing less. Proper diagnosis for restoration by bridge work should include a very careful consideration of the conditions demanding restoration. The proper positions for anchorages, and the force which they will be called upon to bear; the possibility of subsequent danger to such anchorage teeth; the possibility of affecting their vitality or, in the event of necessary devitalization, the proper treatment of the pulp canals; the care as to correct shaping of the walls of the anchorage teeth; the advisability of the use of a fixed or removable type of bridge. If the former, whether or not the use of replaceable porcelain teeth is expedient; the choice between the so-called self-cleansing type of bridge work and a contoured cast base; the indications for the use of cast gold inlays as abutments; the inadvisability of the excessive use of gold shell crowns, especially in the anterior portion of the arch. Any one of these themes is sufficient for a long dissertation, and yet they are positively necessary to consider if we are to conscientiously give to our patients the best restoration for the needs of each. In the light of the development made along the lines of oral hygiene, both within the profession and among the laity, the introduction of many of our bridges constructed as they have been in the past, will not be permitted. So little forethought has been given to the dynamics of bridge work that it is not to be wondered at that even well made bridges have failed, after a few years service. When we consider how much is expected of an anchorage tooth, beyond its normal functional burden, we should not willfully shorten its life by adding to its load. Many bridges of the fixed type have done, and are doing, excellent service, but many others, had they been of the removable variety, would have been less a burden upon the anchorages. There are so many methods offered for this means of attachment that it seems probable that within a few years all bridge work will be of the removable type. This will furnish restorations which will be possible for the patient to cleanse as carefully as he does his natural teeth. It will also afford opportunities for placement of a large share of the burden where it belongs —upon the ridge, and not upon the anchorage teeth—thus lightening materially the load.

It will furnish opportunity to the operator to make better facial contour restoration than is possible with many types of fixed bridgework. By the use of the casting processes, extension saddles may be provided which will rest absolutely firm upon the ridge. These saddles, in combination with any of the various types of replaceable porcelain teeth result in a restoration which is both natural and useful for masticatory purposes.

One field of our prosthetic work has been woefully put to one side, probably because of its expensive character, owing to its base of platinum, and that is continuous gum work. No restoration for edentulous cases can compare with it and the possibilities to be obtained by the artistic arrangement of the teeth, including the individual treatment of each tooth, and the natural contouring and enameling of the gum.

